# Expression of ACE2, Soluble ACE2, Angiotensin I, Angiotensin II and Angiotensin-(1-7) Is Modulated in COVID-19 Patients

**DOI:** 10.3389/fimmu.2021.625732

**Published:** 2021-06-14

**Authors:** Ikram Omar Osman, Cléa Melenotte, Philippe Brouqui, Matthieu Million, Jean-Christophe Lagier, Philippe Parola, Andréas Stein, Bernard La Scola, Line Meddeb, Jean-Louis Mege, Didier Raoult, Christian A. Devaux

**Affiliations:** ^1^ Aix-Marseille Univ, IRD, APHM, MEPHI, IHU-Méditerranée Infection, Marseille, France; ^2^ Aix-Marseille Univ, IRD, APHM, SSA, VITROME, IHU-Méditerranée Infection, Marseille, France; ^3^ Centre National de la Recherche Scientifique (CNRS), Marseille, France

**Keywords:** COVID-19, ACE2 (Angiotensin Converting Enzyme-2), Renin – Angiotensin – Aldosterone System, SARS – CoV – 2, Hypertension

## Abstract

The etiological agent of COVID-19 SARS-CoV-2, is primarily a pulmonary-tropic coronavirus. Infection of alveolar pneumocytes by SARS-CoV-2 requires virus binding to the angiotensin I converting enzyme 2 (ACE2) monocarboxypeptidase. ACE2, present on the surface of many cell types, is known to be a regulator of blood pressure homeostasis through its ability to catalyze the proteolysis of Angiotensin II (Ang II) into Angiotensin-(1-7) [Ang-(1-7)]. We therefore hypothesized that SARS-CoV-2 could trigger variations of ACE2 expression and Ang II plasma concentration in SARS-CoV-2-infected patients. We report here, that circulating blood cells from COVID-19 patients express less ACE2 mRNA than cells from healthy volunteers. At the level of circulating cells, this *ACE2* gene dysregulation mainly affects the monocytes, which also show a lower expression of membrane ACE2 protein. Moreover, soluble ACE2 (sACE2) plasma concentrations are lower in prolonged viral shedders than in healthy controls, while the concentration of sACE2 returns to normal levels in short viral shedders. In the plasma of prolonged viral shedders, we also found higher concentrations of Ang II and angiotensin I (Ang I). On the other hand, the plasma levels of Ang-(1-7) remains almost stable in prolonged viral shedders but seems insufficient to prevent the adverse effects of Ang II accumulation. Altogether, these data evidence that the SARS-CoV-2 may affect the expression of blood pressure regulators with possible harmful consequences on COVID-19 outcome.

## Introduction

Similarly to the severe acute respiratory syndrome coronavirus (SARS-CoV) that emerged in Asia in 2003, SARS-CoV-2 is a *Betacoronavirus* lineage 2b/*Sarbecovirus* which found its origin in a coronavirus circulating in wildlife and to which humans are susceptible (1, 2). The SARS-CoV and SARS-CoV-2 are genetically close and shared 79.5% nucleotide identity (3–6). SARS-CoV-2, is the etiological agent of coronavirus disease 2019 (COVID-19). First described in Chinese patients hospitalized in December 2019, COVID-19 cases were then reported in many countries and the original virus as well as its variants have actually spread worldwide (6–8). Although not very symptomatic for the majority of those infected, the SARS-CoV-2 can be responsible for severe forms of COVID-19 characterized by multiple organ dysfunction syndrome (MODS) (9, 10) as well as acute respiratory distress syndrome (ARDS), with high fatality risk (11). In 16 months, the COVID-19 pandemic caused more than 3.4 million deaths and 164 million confirmed cases - (the COVID-19 Dashboard at Johns Hopkins University May 18, 2021: https://coronavirus.jhu.edu/map.html).

Venous thromboembolism is a relatively common side effect of SARS-CoV-2 infection (up to one-third of critical COVID-19 cases), characterized by acute pulmonary embolism or intravascular coagulopathy that predisposes the patients to thrombotic events (12–14). Elevated D-dimers upon admission of patients is a biomarker of pulmonary embolism and is associated with increased mortality (15). Cytokines (such as IL-6) production is increased in severe COVID-19 and contributes in the “cytokine storm” leading to obstructive thrombo-inflammatory syndrome (16–18). Anticoagulants are considered a possible therapy to reduce the harmful circle of inflammation-coagulation-fibrosis observed in severe forms of COVID-19 (19, 20). To further explore the physiopathology of COVID-19, it is necessary to focus research on the viral receptor, the ACE2 molecule, and its function. The ACE2 expressed at the surface of human alveolar pneumocytes is the receptor for the spike proteins (S1) of SARS-CoV-2 (21–23). Cell surface expression of ACE2 is however not limited to pneumocytes as the *ACE2* gene is differentially expressed in human tissues, offering a broad spectrum of cellular targets to the virus including enterocytes and the arterial and venous endothelial cells (24–27).

The interaction between S1 and ACE2 does not solely allow viruses attachment to target cells, it could possibly disturb the function of ACE2. Indeed, ACE2, a 805 amino acids type I cell-surface zinc-dependent monocarboxypeptidase, catalyzes the hydrolysis of the active vasoconstrictor octapeptide Ang II [Asp-Arg-Val-Tyr-Ile-His-Pro-Phe] to the heptapeptide Ang-(1-7) and free L-Phe, thus controlling blood pressure (28–31). Therefore, ACE2 antagonizes the vasoconstrictor and profibrotic effects of Ang II both by reducing the synthesis of Ang II and by catalyzing the transformation of Ang II into Ang-(1-7) (32). ACE2 can also exert its functions through cleavage of Ang I (the precursor of Ang II) into Ang-(1-9), which can get further metabolized into Ang-(1-7) (33). Characterizing possible dysregulation of the renin angiotensin aldosterone system (RAAS) in SARS-CoV-2 infected patients, could help explain why one of the main comorbidities related to COVID-19 is hypertension (HT) (34, 35). It may also encourage the use of new classes of molecules such as soluble human recombinant ACE2 (shrACE2) (36), in the treatment of COVID-19. Indeed, it has recently been reported that the administration of shrACE2 to severe COVID-19 patients quickly improved their condition (37).

It has previously been reported that SARS-CoV, which uses ACE2 for binding to cells as does SARS-CoV-2, modulates the expression of ACE2 (38–40). We have recently hypothesized (41) that a SARS-CoV-2-induced decrease in ACE2 expression and/or inhibition of ACE2 peptidase function could result in a feedback control leading to increased production of Ang II expected to aggravate the severity of COVID-19. To investigate this hypothesis we studied the expression of ACE2 mRNA, ACE2 cell-surface protein, as well as the plasma concentrations of soluble ACE2 (sACE2), Ang I, Ang II, and Ang-(1-7), in 44 patients (30 prolonged viral shedders - and 14 short viral shedders) compared to 15 healthy volunteers. We found here that the concentrations of these biomarkers are significantly affected during COVID-19 and we suggest that these changes are likely conducive to worsening the patient’s condition.

## Materials and Methods

### Study Population

This prospective pilot study included 44 patients with a confirmed diagnosis of SARS-CoV-2 infection diagnosed at the IHU Méditerranée Infection in Marseille. These patients have all been tested positive for SARS-CoV-2 in their nasopharyngeal samples as evidenced by a quantitative reverse transcriptase-polymerase chain reaction (qRT-PCR) which uses specific primers for the E gene of this virus. The qRT-PCR were considered positive when the cycle threshold (Ct) was below 30, as previously reported (42). The severity of the disease was determined according to the WHO classification (43). Before inclusion, the patients were all subjected to a clinical examination including a pulmonary examination by low dose scanner and their blood pressure was recorded. The inclusion period began on 3 June 2020 and continued until the authorized number of patients was reached. Patients were distributed in two groups: i) prolonged viral shedders (nasopharyngeal SARS-CoV-2 PCR ≥10 days from the first consultation) and, ii) short viral shedders (nasopharyngeal SARS-CoV-2 PCR<10 days from the first consultation). A control group was composed with healthy volunteers. All patients included in this study received 600 mg of hydroxychloroquine (HCQ) daily during 10 days (plus or minus 1 day) and azithromycin was added to their treatment during the first 5 days. This therapy, routinely used at the IHU Méditerranée Infection, as previously described (44, 45), was initiated with the informed consent of the patient and after verification of the absence of contraindications. A follow up of the patients’ viral load was performed to monitor the effectiveness of treatment and the course of the disease at days 0, 3, 6 and 10. Deidentified clinical information was collected from the standard clinical care units of the IHU Méditerranée Infection, in accordance with regulations in force in France. Most of the patient samples used in this study were collected between 1 and 3 days after the treatment had been stopped (with exception of two samples collected 5 days after the end of treatment, and samples from 2 other patients collected 7 days and 10 days respectively after the end of treatment). Samples (10 mL Paxgene tube and 5mL EDTA tube), were collected and divided into the predefined two patients groups and control group, respectively. All healthy volunteers (students and non-nursing staff with no symptoms) were tested negative for SARS-CoV-2 by qRT-PCR (Ct>34).

### Ethical Approval

This research protocol registered as ANSM N° 2020-a00864-35, was submitted to ethical review by the French Participants Protection Committee (CPP East III; CPP president: Dr. P. Peton) - and received ethical approval prior to participant’s enrolment (CPP 20.04.09/N°:20.04.01.83219/N°7626; Principal investigator, C. Devaux, Research Director at CNRS; Assay promotor: IHU Méditerranée Infection: Prof Didier Raoult, Director). All patients were informed of the nature of the study and provided written consent. The volume of peripheral blood sampled agreed with the volume authorized in type 2 human biological research (RIPH type 2) and was approved by the ethics committee for this study. All clinical data were deidentified before being made available to the principal investigator and collaborators in respect with the European General Data Protection Regulation (GDPR).

### Plasma Collection

5 mL of blood samples was collected from patients and healthy donors into an Ethylenediaminetetraacetate (EDTA) tube (Sigma Aldrich, Saint-Quentin Fallavier, France), and allowed to clot at room temperature. The plasma was collected, then centrifuged at 1500g for 10 min and stored at -20°C until use.

### PBMCs and Adherent Cells (Monocytes) Isolation

Peripheral blood mononuclear cells (PBMCs) were recovered from blood samples using Ficoll (Eurobio, Les Ulis, France) density gradient centrifugation followed by harvesting of PBMCs. Monocytes were isolated by adherence to culture dishes for 2 hours at 37°C and directly lysed for qRT-PCR without being harvested.

### Ribonucleic Acid (RNA) Extraction and Quantitative-Reverse Transcription Polymerase Chain Reaction (qRT-PCR)

RNAs were extracted from whole blood using PAXgene blood RNA kit (QIAGEN SA) with a DNase I step to eliminate DNA contaminants, according to the manufacturer instructions. The quantity and quality of the RNA was evaluated using a Nanodrop 1000 spectrophotometer (Thermo Science). The first-strand cDNA was obtained using oligo(dT) primers and Moloney murine leukemia virus-reverse transcriptase (MMLV-RT kit; Life Technologies), using 200 ng of purified RNA. The amplification cycles were performed using a real time PCR Mastercycler gradient (Eppendorf, Montesson, France). The qPCR experiments were performed using specific oligonucleotide primers (Specific primers were designed using the Primer3 software) and hot-start polymerase (SYBR Green Fast Master Mix; Roche Diagnostics). The specific primers used were Angiotensin Convertase Enzyme (ACE2) primers (forward primer: CAGGGAACAGGTAGAGGACATT and reverse primer: CAGAGGGTGAACATACAGTTGG) and the housekeeping β-Actin primers (forward: AGGAAGGAAGGCTGGAAGAG and reverse: GGAAATCGTGCGTGACATTA). The amplification cycles were performed using a C1000 Touch Thermal cycler (Biorad, Marnes-La-Coquette, France). The results of qRT-PCR were normalized using the housekeeping gene β-Actin (ACTB) and expressed as Relative Quantity (RQ= 2^-ΔCT^), where ΔCt = (Ct_Target_ − Ct_Actin_). The threshold cycle (Ct) was defined as the number of cycles required to detect the fluorescent signal.

### Quantification of Plasma solubleACE2, Angiotensin I, Angiotensin II, and Angiotensin-(1-7)

The quantity of soluble ACE2, Angiotensin I, Angiotensin II, and Angiotensin-(1-7) in the plasma of healthy volunteers and COVID-19 patients was determined using Elisa kits (FineTest, Wuhan, China) that require small sample volume and obviate the need for prior sample extraction or enrichment. Plasma samples were rapidly processed and kept at -20°C until used, as suggested by Chappell et al. (46, 47). All studied samples were tested on the same day. The experiments were performed according to the manufacturer’s instructions, using 100 µL of plasma per assay. The minimal detectable concentration of human soluble ACE2, Angiotensin I, Angiotensin II, Angiotensin-(1-7) were 391 pg/mL, 125 pg/mL pg/mL, 31.2 pg/mL, and 15.6 pg/mL, respectively. The concentrations in each molecule were calculated by comparison to standard curves.

### Flow Cytometry

Flow cytometry was used to study the membrane expression of ACE2 as well as specific biomarkers on PBMCs. Cells were analyzed according to fluorescence intensity using an anti-ACE2-monoclonal antibody (mAb) labeled with Alexa Fluor 488 (R&D Systems, Minneapolis, USA) and mAb anti-CD3-PC5, anti-CD20-PC7, anti-CD16-PE, and anti-CD14-APC purchased from Beckman (Beckman coulter, Villepinte, France). Fluorescence intensity was measured using a Canto II cytofluorometer (Becton Dickinson, Biosciences, Le Pont de Claix, France) and the results were analyzed using a BD FACSDiva software v.6. 1.3 (Becton Dickinson, New Jersey, USA).

### Statistical Analysis and Correlation Analysis

Data were analyzed using a non-parametric Mann-Whitney U test to compare healthy donors, patients with COVID-19 and healed COVID-19 individuals. The results are presented as the median with confidence interval. A p value <0.05 was considered statistically significant. The data were submitted to multivariate principal component analysis (PCA) biplot (score plot + loading plot) (RStudio) and hierarchical clustering heatmap analysis (ClustVis). The R studio program (RStudio, USA) was used to generate the correlation matrix for the biomarkers of interest after a Pearson’s test that measuring the linear correlation for two variables or Spearman’s test for the not normally distributed correlation.

## Results

### Characteristics of Patients

All COVID-19 patients included had mild to moderate disease and recovered from COVID-19 infection. Patients were divided into two groups. The first group, hereafter named prolonged viral shedders (patients with viral persistence 10 days after diagnosis of infection), was composed of 30 patients having difficulties to clear the virus, among whom 5 men and 25 women (mean age: 46 ± 13 years). The second group, hereafter named short viral shedders (patients with rapid viral clearance at day 6 after diagnosis of infection), included 14 patients -, among whom 7 men and 7 women (mean age: 41 ± 12 years) (**Table 1**). Most samples (40/44) were collected between 11 and 13 days after SARS-CoV-2 diagnosis (i.e. less than 5 days after the test turned negative on the nasopharyngeal sample for the prolonged viral shedders and more than 5 days after qRT-PCR turned negative on nasopharyngeal sample for the short viral shedders group), whereas 4 samples were collected between 15 and 20 days post-diagnosis due to the unavailability of patients. A group of 15 healthy volunteers was included in this study as control among which 7 males - and 8 females (mean age: 34.8 ± 14 years).

**Table 1:** Patients/clinical data.

Clinical characteristics	Prolonged viral shedders (nasopharyngeal SARS-CoV-2 shedding ≥10 days) N=30	Short viral shedders (nasopharyngeal SARS-CoV-2 shedding <10 days) N=14	Healthy volunteers (nasopharyngeal SARS-CoV-2 negative) N=15
Sex ratio (M/F)	5/25	7/7	7/8
Age (Mean ± SD)	46 ± 13	41 ± 12	34.8 ± 14
Comorbidities			
-Arterial hypertension	4	2	0
-Diabetes melitus	1	1	0
-Diabetes type 1	0	1	0
-Unique kidney	0	1	0
-Pituitary adenoma	0	1	0
-Obesity	2	0	0
-Arrythmia	2	0	0
Anti hypertensive treatment/cardiac therapy	1	0	0
-Sartan	1	0	0
-Beta-blocking	3	0	0
-Calcic inhibitors	1	0	0
-Flecain	1	0	0
Other medication			
-Tryptan	1	0	0
-levothyrox	1	0	0
-ostro-progestatuve	1	0	0
-Ventoline	1	0	0
-Beclometasone/formoterol inhalated	1	1	0
-Paroxetine/duoxetine	2	0	0
-Insuline	0	1	0
SARS-CoV-2 infection			
-Positive qRT-PCR (Ct**mean ± SD)	100% (21.7 ± 5)	100% (24 ± 8.1)	0
-Serology (positive/tested)	13/28	8/13	0/15
Potassium			
High >4.5 mmol/L	2	2	NA*
Low <3.5 mmol/L	4	0	NA
Delay between the diagnosis and sampling (days)	14 ± 2.9	13 ± 4.2	NA

*NA, Not applicable.

**Ct, Cycle threshold (positive qRT-PCR: Ct <30).

#### Lower ACE2 mRNA Expression in Circulating Blood Cells From COVID-19 Patients

**Figure 1:**
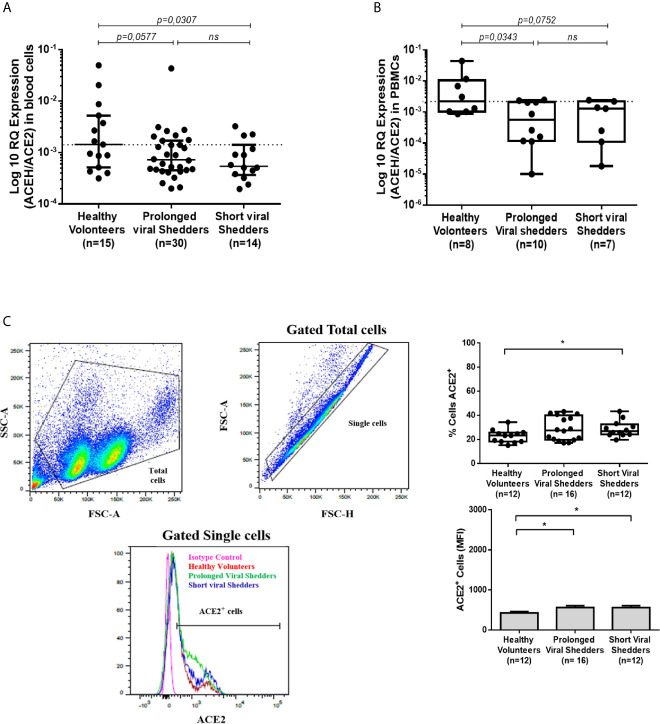
ACE2 mRNA expression in blood cells and PBMCs, and monocytes from COVID-19 patients and healthy volunteers. **(A)** Expression of ACE2 mRNA in circulating blood cells (both mononuclear and polynuclear cells) from the prolonged viral shedders (n = 30) and the short viral shedders (n = 14) groups, compared to a healthy volunteers control group (n= 15). The results (presented as median with confidence interval) are expressed as log10 RQ were RQ = 2^(−ΔCT)^. **(B)** Expression of ACE2 mRNA in peripheral blood mononuclear cells (PBMCs) samples from the three different groups studied: prolonged viral shedders (n = 10), short viral shedders (n = 7), and healthy volunteers (n= 8). The data are expressed as log10 RQ were RQ = 2^(−ΔCT)^. **(C)** Flow cytometry analysis of ACE2 expression at the surface of total blood cells from prolonged viral shedders (n=16) and short viral shedders (n=12) versus total blood cells from healthy volunteers (n=12). The left panels show the gating parameter chosen (from individual to individual, between 8 and 40% of the total blood cells express ACE2 at their surface); the upper right panel indicates the percent of cells expressing ACE2 while the lower right panel is an histogram representing the mean fluorescence intensity (MFI) The Mann–Whitney test was used for the statistical analysis of all the data. The result was considered significant when the p value < 0.05: *; ns for not significant result.

In order to assess the effect of SARS-CoV-2 on the *ACE2* gene expression in patients with COVID-19, we used qRT-PCR to compare the ACE2 mRNA expression in circulating cells from 30 prolonged viral shedders and 14 short viral shedders with 15 healthy volunteers (**Figure 1A**). When the median values of ACE2 mRNA expression were compared among groups, a lower expression of ACE2 mRNA was observed in the prolonged viral shedders compared to the healthy volunteers (median values 0.722 x 10^-3^ versus 1.433 x 10^-3^). Yet, this result appeared not statistically significant according to Mann-Whitney test (p=0.0577). This lower ACE2 gene expression was also observed in the short viral shedders compared to the healthy volunteers (median values 0.540 x 10^-3^ *versus* 1.433 x 10^-3^), and even more accentuated then in the prolonged viral shedders group. The lower expression of ACE2 mRNA in short viral shedders was statistically significant - (p=0.0307). In addition, there were no significant differences between the two groups of patients (p=0.2771). We confirmed these results by investigating the expression of ACE2 mRNA in PBMCs, instead of the whole blood circulating cells (the difference resides in the lack polynuclear cells in PBMCs) (**Figure 1B**). Out of the 44 people from the patients’ groups, 17 patients were tested (we had not enough cells in all samples collected to test the 44 patients). Under these new experimental conditions, the results were very similar to the previous ones with a lower ACE2 mRNA expression in the two groups of COVID-19 patients - compared to healthy volunteers (median values 0.561 x 10^-3^ and 1.28 x 10^-3^ versus 2.18 x 10^-3^ for the prolonged viral shedders, the short viral shedders, and the healthy volunteers, respectively). This lower ACE2 gene expression in the prolonged viral shedders group (n=10), was found significant compared to the group (n=8) of healthy volunteers (p=0.0343). Although a comparison of the median values of short viral shedders with those in the healthy volunteers group (median values 1.28 x 10^-3^ versus 2.18 x 10^-3^), showed a slightly lower ACE2 gene expression, this result is not statistically significant (p=0.0752) for this second group of patients.

Since ACE2 mRNA expression was found lower in circulating blood cells from COVID-19 - patients, we investigated whether this could be correlated with the amount of ACE2 protein expressed at the surface of PBMCs. To this end, after incubation with Alexa Fluor 488-labeled anti-ACE2 mAb, PBMCs from COVID-19 patients and healthy volunteers were examined by flow cytometry. Between 8 and 40% of PBMCs expressed detectable ACE2 at their surface. ACE2 expression at the surface of PBMCs from the prolonged viral shedders (n=16) and the short viral shedders (n=12), was quantified and compared to that of PBMCs from the healthy volunteers (n=12) (**Figure 1C**). Surprisingly, the percentage of PBMCs expressing membrane-bound ACE2 was almost stable or even slightly higher in the two groups of patients compared to the healthy volunteers.

### Lower ACE2 Protein Expression in CD14^+^/HLADR^+^ Monocytes From COVID-19 Patients

**Figure 2:**
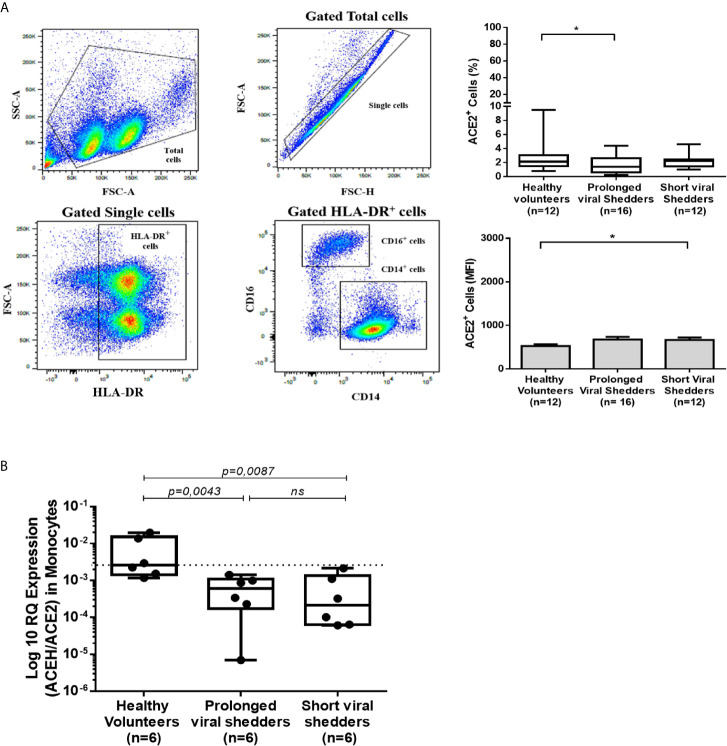
ACE2 expression in HLA-DR^+^/CD14^+^ monocytes from COVID-19 patients and healthy volunteers. **(A)** Flow cytometry analysis of ACE2 protein expression at the surface of different cell populations during COVID-19. The figure illustrates the results obtained with the HLA-DR^+^/CD14^+^ population while data obtained with respect to other cell populations are presented in the **Supplementary Figure S1**. The gating (left panels) was performed using different cluster differentiation-specific mAb (CD14, HLA-DR). The right panels show the percent of monocytes expressing ACE2 (upper right panel) and their ACE2 mean fluorescence intensity (lower right panel). **(B)** Analysis of ACE2 mRNA expression in monocytic cells (simply selected by adherence to tissue culture plates) using qRT-PCR. Monocytic cells from prolonged viral shedders (n = 6), short viral shedders (n = 6), and healthy donors (n = 6), were tested for ACE2 mRNA expression. The data are expressed as log10 RQ were RQ = 2^(−ΔCT)^. Mann–Whitney test was used for the statistical analysis of all data. *p value < 0.05; ns, not significant.

Although ACE2 mRNA expression was lower in PBMCs from COVID-19 patients than in PBMCs from healthy volunteers, this does not result in a lower expression of the ACE2 protein on the total population of PBMCs. To try to understand this apparent paradox, the expression of ACE2 on the surface of separated cell populations was studied. The modulation of ACE2 cell-surface expression differed from one cell population to another as illustrated by the significantly higher number of CD3^+^ T cells expressing ACE2 in the COVID-19 patients groups, while there was an absence of modulation in the CD20^+^ B cells and CD16^+^/HLA-DR^+^ monocytic/dendritic cells expressing ACE2 compared to cell populations from healthy volunteers (**Figure S1** **;** **Supplementary Data**). However, we observed (**Figure 2A**) a slight but significantly lower number of CD14^+^/HLA-DR^+^ monocytes that expressed membrane-bound ACE2 in the prolonged viral shedders (n=16). This lower expression of ACE2 in monocytes was confirmed using qRT-PCR (**Figure 2B**). A significantly lower ACE2 mRNA expression - was found in monocytic cells from the prolonged viral shedders (n=6; p=0.0043) and the short viral shedders (n=6; p=0.0087), compared to the same cell type in healthy volunteers (n=6).

### Lower Plasma Concentrations of sACE2 in COVID-19 Patients

**Figure 3:**
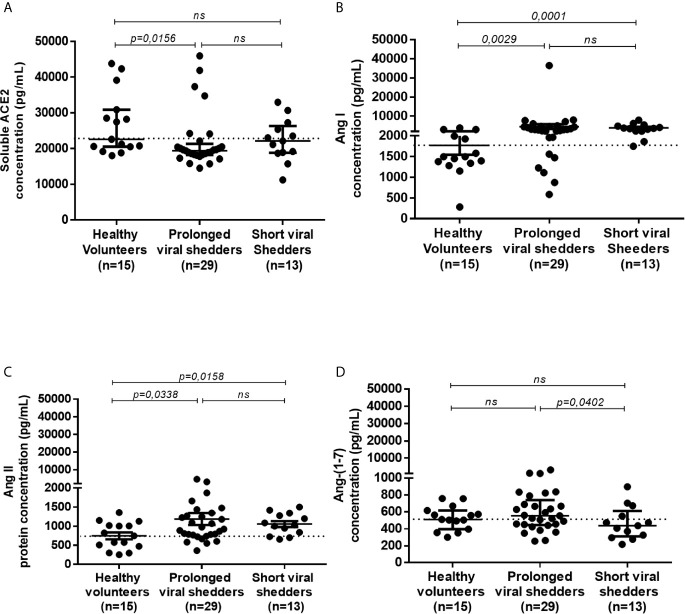
ELISA quantification of the sACE2 protein, and angiotensin metabolites Ang I, Ang II, and Ang-(1-7), in different groups of COVID-19 patients and healthy volunteers. Quantification of sACE2 **(A)**, Ang I **(B)**, Ang II **(C)**, and Ang-(1-7) **(D)** in the plasma of prolonged viral shedders (n=29) and short viral shedders (n=13) compared to the healthy volunteers control group (n=15), using antigen-specific ELISA. The results are presented as median with confidence interval. The Mann–Whitney test was used for the statistical analysis. ns, not significant.

The release of soluble ACE2 (sACE2) in plasma was investigated using an ELISA assay (**Figure 3A**). Plasma samples from two patients were not available; thus, only 29/30 patients from the prolonged viral shedders group and 13/14 patients from the short viral shedders group were tested. The expression of sACE2 was found heterogeneous among the healthy control group. In the healthy controls group, 3 individuals were found with higher concentrations of sACE2 than the other group members, among which a 32 years old man characterized by a high expression of ACE2 mRNA. In the prolonged viral shedders, there was also a strong heterogeneity with 5 patients - for whom the expression was much higher than for the 25 other members of the group. All 5 patients were women, including two with high blood pressure, but none of them had elevated expression of ACE2 mRNA. Regarding the short viral shedders, the results were more homogeneous. Despite these individual variations, what is remarkable according to a Mann-Whitney test, is that the plasma concentration of sACE2 was found to be significantly lower in the prolonged viral shedders, compared to the healthy volunteers’ group (19396 pg/mL versus 22600 pg/mL; p=0.0156). It should also be noted that the short viral shedders had a plasma concentration of sACE2 of 22141 pg/mL, which was not significantly different from the healthy control group (p=0.153).

### Higher Plasma Concentrations of Ang I and Ang II in COVID-19 Patients

Since ACE2 is a key peptidase that regulates the - RAAS, it was of major importance to investigate the plasma concentration of the main peptides of this system. The quantification of Ang I, a peptide resulting from the cleavage of the angiotensinogen by renin was performed as it serves as upstream precursor for Ang II production following hydrolysis by the ACE peptidase. In addition, since ACE2 mRNA expression was found to be lower in patients with COVID-19, it was of interest to study whether this modification affected the plasma concentrations of Angiotensin metabolites. For this purpose, we used Ang I and Ang II ELISA kits. The comparison of Ang I concentrations found in healthy volunteers (median=1496 pg/mL), with that from COVID-19 patients showed a significantly higher Ang I in both prolonged viral shedders (median= 2979 pg/mL; p=0.0029) and short viral shedders (median= 3603 pg/mL; p=0.0001) (**Figure 3B**). Next, Ang II was quantified in the patient’s plasma and that of the healthy volunteers. This study showed that the - Ang II concentration was higher in the prolonged viral shedders (median= 921 pg/mL; p=0.0338) and short viral shedders (median= 1038 pg/mL; p=0.0158), than in the healthy volunteers (median=746 pg/mL) (**Figure 3C**). The differences between the two groups of patients and the healthy volunteers were found to be significant according to a Mann-Whitney test - It is noteworthy that the plasma concentration of Ang II was extremely high in two patients from the prolonged viral shedders group, two women (49 years old and 54 years old, respectively) - without documented history of high blood pressure and anti-HT treatment. Their kalemia values were also in the normal range (4.06 mmol/L and 4.1 mmol/L, respectively).

### Stable Plasma Concentrations of Ang-(1-7) in COVID-19 Patients

The finding that Ang II accumulated in the plasma of COVID-19 patients prompted us to study the plasma concentrations of Ang-(1-7), the downstream molecule of the RAAS pathway. We studied the Ang-(1-7) plasma concentrations in the 3 studied groups (**Figure 3D**). Indeed we found a slightly higher plasma concentrations of Ang-(1-7) in the prolonged viral shedders (median: 554 pg/mL) than in the short viral shedders (median: 436 pg/mL). We noted in particular a strong expression of Ang-(1-7) in three prolonged viral shedders, one being the patient with the highest plasma concentration of Ang II in this group, the second patient was a 81 years old woman with HT (candesartan medication; kalemia: 3.68 mmol/L), while the third patient was a 52 years old man with no known history of HT (kalemia: 3.82 mmol/L). However, compared to the Ang-(1-7) plasma concentrations found with samples from healthy volunteers (median: 510 pg/mL), the higher Ang-(1-7) concentrations measured in samples from prolonged viral shedders was not statistically significant.

### Principal Component Analysis and Hierarchical Clustering of Data

**Figure 4:**
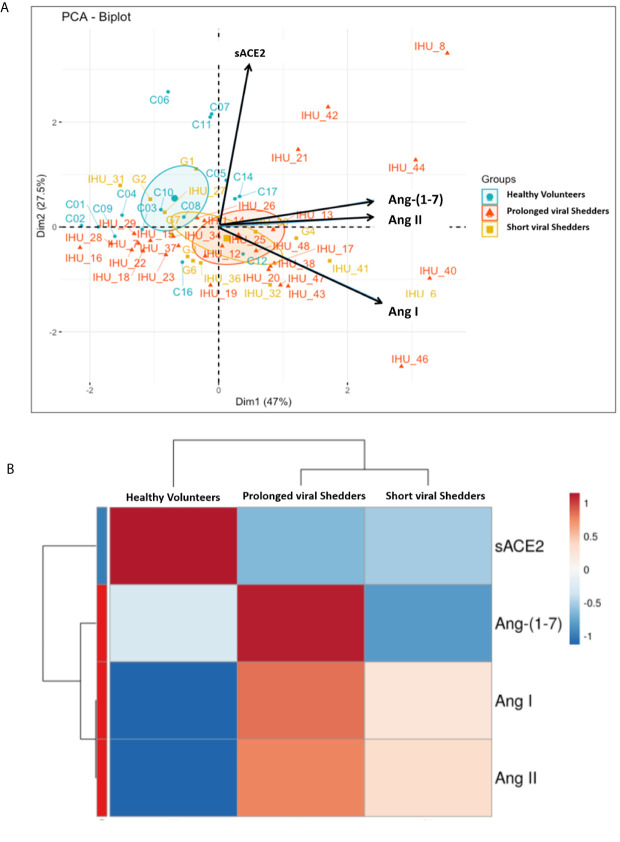
**(A)** Representation of the principal component analysis (PCA) biplot (score plot + loading plot). All patient codes and clinical results were deidentified before being made available to the principal investigator **(B)** Hierarchical clustering heatmap analysis (each colored cell on the map corresponds to a concentration value) of the different angiotensin metabolites in each group.

Regarding the differences of angiotensin metabolites concentrations between the two groups of patients and the healthy volunteers they were found to be significant according to a Mann-Whitney test and were also confirmed by the principal component analysis and hierarchical clustering heatmap (**Figure 4** and **Figure S2** **;** **Supplementary Data**) which show that the two groups of COVID-19 patients behave differently from the control group for all parameters studied. In fact, it appears that higher concentrations in Ang I correlate with higher concentrations in Ang-(1-7) (r=0.49, p <0.006 in prolonged viral shedders and r=0.74, p=0.0036 in short viral shedders). Regarding Ang II, we found that higher concentrations in Ang II also correlate with higher concentrations in Ang-(1-7) and this effect was statistically significant (r=0.60, p <0.001 in prolonged viral shedders and r=0.89, <0.001 in short viral shedders). Yet, as shown in **Figure 3D**, we also found a statistically significant decrease in Ang-(1-7) between prolonged viral shedders (median: 554 pg/mL) and short viral shedders (median: 436 pg/mL; p=0.00402).

## Discussion

**Figure 5:**
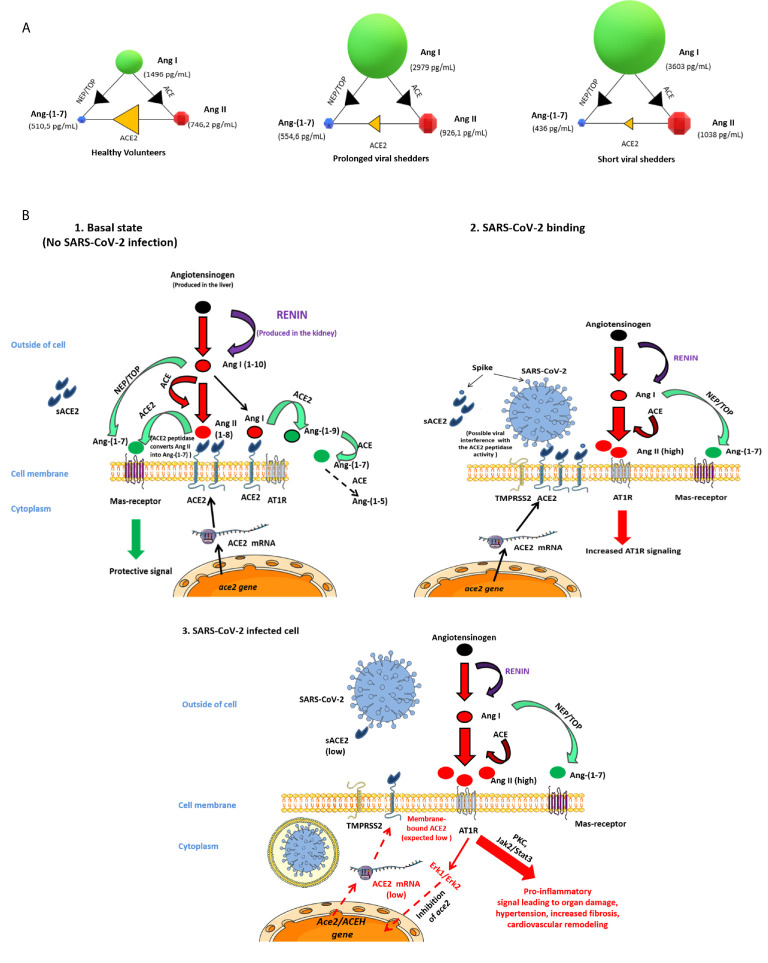
Schematic representation of the possible dysfunction of the angiotensin metabolites pathway in COVID-19 patients. **(A)** Modelization of the concentration of angiotensin metabolites in plasma of healthy volunteers, prolonged viral shedders, and short viral shedders. For each group, the individual circles indicate the metabolite median concentrations, shown in pg/mL. The arrows between the circles mark the catalyzing reactions mediated by the indicated enzymes. **(B)** Schematic diagram of the renin-angiotensin system (RAS) cascade in a normal physiological state and in COVID-19 patients. Renin cleaves Angiotensinogen to Ang I which is further processed to the vasoconstrictor Ang II peptide by ACE. The upper left panel illustrates the Ang II pathway of Ang-(1-7) biosynthesis where ACE2 converts Ang II to Ang-(1-7) and also Ang I to Ang-(1-9) next converted in Ang-(1-7) by ACE peptidase, leading to protective signal through MAS-receptor. This is the physiological state. The upper-right panel illustrates the possible dysfunction of signals when SARS-CoV-2 is attached to its ACE2 receptor. Under this condition Ang-(1-7) synthesis through ACE2 is likely decreased, Ang II accumulates and, increased Ang II binding to AT1R - leads to proinflammatory signals that ultimately will trigger both tissues damage (in particular lungs and heart) and hypertension. The lower - panel illustrates that during COVID-19, the ACE2 mRNA expression is reduced leading to low surface expression of ACE2 (and plasma sACE2), Ang II is in excess in plasma, yet Ang-(1-7) remains synthesized through the NEP/TOP alternative pathway where neprilysin (NEP) and thimet oligopeptidase (TOP) convert Ang I to Ang-(1-7).

During the past year, *in vitro* and *in silico* studies allowed to analyze interactions between SARS-CoV-2 and ACE2 (48–50). This has led many clinical research teams to hypothesize that soon after infection of people, SARS-CoV-2 is likely to trigger a dysfunction of ACE2 and subsequently variations in the balance between Ang II and Ang-(1-7), contributing to worsening hypertension and releasing of proinflammatory cytokines, especially IL-6, thereby accelerating - atherogenesis (35, 51). ACE2 is thought to act in an opposing manner to its homologue, angiotensin‐converting enzyme (ACE), by inactivating the vasoconstrictor peptide Ang II and generating the vasodilator fragment, Ang-(1–7) (52). As illustrated by **Figure 5**, we report here - evidence that ACE2 mRNA and ACE2 protein expression, as well as sACE2, Ang I, Ang II and Ang-(1-7) plasma concentrations are modulated during COVID-19.

Because the size of the studied cohort is small to meet the recommendations of the ethical committee, this impacted our statistical analyses. This work should later be reproduced with a larger cohort of patients, including patients with severe forms of COVID-19. Moreover, in order to conduct this study, we only had access to blood samples, because it is a simple medical act, not particularly invasive, and it can be performed with the informed consent of the patient in agreement with the ethics committee’ instructions. Although the *ACE2* gene is usually considered poorly expressed in immune cells, the expression of ACE2 mRNAs was previously reported in a subset of CD14^+^ CD16^-^ human monocytes (53), opening to us the possibility of conducting a study using circulating blood cells. However, - it should be kept in mind that it is mainly at the level of the pulmonary, vascular, renal, and cardiac epithelial tissues that the impact of the SARS-CoV-2 should be the greatest - (54–56). There is not such limitation for the quantification of plasma compounds (sACE2, Ang I, Ang II and Ang-(1-7). Regarding the cohort of 44 patients we studied, there is a possible bias due to sex ratio, genetic polymorphism, age, and treatment. In our studied, men represented only 16.6% of the COVID-19 patients group while, in general, about two-thirds of symptomatic COVID-19 are men. Moreover, we did not get information on the sequences of the *ACE* and *ACE2* genes of the patients while ACE and ACE2 ethnic polymorphisms were reported (57–62). Regarding the plasma concentration of sACE2, literature reports that males express little higher ACE2 than females (54, 63, 64). In the two groups of COVID-19 patients, among 44 individuals, seven were known to have a history of hypertension and two more were found with high blood pressure at the time of clinical examination. In the prolonged viral shedders group, 4 patients showed hypokalemia while 2 patients showed hyperkalemia, and in the short viral shedders group, 2 patients showed hyperkalemia. Finally, it cannot be excluded that the treatment taken by the patients might modify the ability of the virus to bind to the ACE2 since hydroxychloroquine - was suggested to mediate a deficit in the glycosylation of ACE2 (65, 66).

Despite the high heterogeneity of ACE2 mRNA expression from individual to individual within each group, we observed a - lower ACE2 mRNA expression in both COVID-19 patient groups compared to the healthy volunteers. It is worth noting that the level of ACE2 mRNA expression in the healthy volunteers group was highly heterogeneous, particularly regarding three volunteers, a 63-years-old male, a 26-years-old female, and a 32-years-old male, who had high basal ACE2 mRNA expression. The difference in ACE2 mRNA expression in peripheral blood cells was significant between the healthy control group and the short viral shedders (p=0.0307), as if the expression of ACE2 retained the imprint of the infection for some time after nasopharyngeal SARS-CoV-2 qRT-PCR has turned negative. In the COVID-19 patient groups, one patient - with an ACE2 expression significantly higher than the mean value for this group was a 47-years-old obese woman on analgesic therapy. Her COVID-19 history was marked by an extended viral carriage during 21 days. In the group of short viral shedders, the three patients with the slightly higher ACE2 mRNA expression were male patients, a 57-years-old patient with hypertension (196/85) and hyperkalemia (4.65 mmol/L) a 66-years-old patient also with above-normal blood pressure (159/95) and a normal kalemia of 3.97 mmol/L, both patients were on anti-HT treatment, - and a 35-year-old male patient (kalemia: 3.91 mmol/L), treated for allergy and inflammatory bronchial obstruction. However, the median value of ACE2 mRNA expression in those patients groups remained lower than the median value of ACE2 mRNA expression in the healthy control group, which is consistent with earlier data obtained *in vitro* - with Sarbecovirus (39). In the absence of Sarbecovirus infection, reduced levels of ACE2 expression were previously reported in cardiac tissues linked to HT, dyslipidemia and/or heart failure (67, 68). The lower ACE2 mRNA expression observed in COVID-19 patients could also be the result of a feedback regulation loop orchestrated by Ang II. It was previously reported that the over-expression of Ang II decreased ACE2 mRNA production in rat cardiac myocytes and fibroblasts (69). This ACE2 mRNA down-regulation was blocked by losartan, an Ang II receptor blocker (ARB), and by inhibitors of MAP kinases, suggesting that Ang II triggers AT1 receptor signaling that leads to Erk1/Erk2 activation and modulation of ACE2 gene expression (69).

During the quantitative analysis of membrane ACE2 by flow cytometry, no decrease in expression of this molecule was observed on PBMCs and populations of T-cells, B-cells and CD16^+^ monocytic/dendritic cells from COVID-19 patients. This was surprising given that there is a decrease in the expression of the ACE2 transcript in PBMCs of COVID-19 patients. It may be due to a lower sensitivity of flow cytometry compared to qRT-PCR. In addition, the mean expression of ACE2 on the surface of PBMCs does not exceed 35%, which is perhaps too low to measure detectable variations at the protein level. Yet we found a significantly lower membrane-bound ACE2 in a population of CD14^+^/HLA-DR^+^ monocytes from prolonged viral shedders, that corroborates with the down regulation of ACE2 transcription in monocytic cells. It is consistent with a previous report indicating that in symptomatic COVID-19 patients the percentage of CD14^+^/HLA-DR^+^ monocytes increase in the PBMCs while the CD14^low^CD16^+^ inflammatory monocytes decrease as they leave the bloodstream and homing tissues (70). Moreover, we found lower plasma concentrations of sACE2 in the prolonged viral shedders consistent with the reduction of ACE2 mRNA expression. Indeed, similarly to other Sarbecoviruses (71, 72) it can be hypothesized that the binding of the SARS-CoV-2 spike to cell-membrane anchored ACE2 modulates ACE2 expression. In a murine animal model, it was observed that Ang II-mediated decrease of ACE2 at the surface of cardiomyocyte was accompanied by the concomitant increase in cell-surface expression of the ADAM17 sheddase (73, 74), suggesting a possible cleavage of membrane-bound ACE2 and shedding of sACE2. It was also previously reported that a recombinant SARS-CoV expressing the HCoV-NL63 spike, another human coronavirus using ACE2 for cell entry, trigger shedding of sACE2 (75). The sACE2 molecule could be considered a possible candidate for monitoring the evolution of COVID-19.

Here we found that the plasma concentrations of Ang I and Ang II are significantly higher in the COVID-19 patients - than in healthy volunteers. It might have seemed reasonable to hypothesize that if, in COVID-19 patients, ACE2 expression was decreased and the Ang II plasma concentration became high, the risk of hypertension could be high. In the prolonged viral shedders group, 7 out of 30 patients had a history of HT and 4 showed evidence of HT during the clinical examination. Two cases (2/23, 8.7%) of HT were discovered at the time of their clinical examination (a patient with blood pressure of 180/30 and hypokalemia at 3.45 mmol/L, and another with blood pressure of 137/65 and a kalemia of 4.1 mmol/L). In the short viral shedders group,- 2 - patients had HT but they were already known for HT prior to the discovery of their COVID-19. It is worth noting that both Ang I and Ang II remained very high in plasma samples of short viral shedders even after the specific SARS-CoV-2 qRT-PCR on nasopharyngeal samples has turned negative. The medical management of hypertension involves the use of RAAS inhibitors, such as angiotensin converting enzyme inhibitors (ACEis) and Ang II Receptor Blockers (ARBs), considered to up-regulate the cell surface expression of ACE2, with the possible adverse effects of increasing the susceptibility to SARS-CoV-2 of patients who receive these treatments (76, 77). Recently we found that ARBs (azilsartan, eprosartan, irbesartan, olmesartan, losartan, telmisartan, and valsartan) trigger a down-regulation of the AT1R mRNA and an over-expression of ACE2 mRNA synthesis in Vero E6 cells that correlates with increased SARS-CoV-2 production (78). However, using a model of mice treated with captopril (ACEi) or telmisartan (ARB), Wysocki and colleagues reported a profound decrease in kidney ACE2 protein and increase in cytosolic ACE2 protein, but this effect was not found in lung (79). Nonetheless, another study reported that ACEis reduced ACE2 expression in lungs (80). In previous opinion papers, we and others (32, 76, 81–83) strongly encouraged to rapidly evaluate whether the - RAAS inhibitors are more beneficial than harmful in severe COVID-19 patients. This question was - the source of intense debate among cardiologists - (41, 74, 84), with supporters of the temporary discontinuation of the treatment during COVID-19 to avoid viral over-replication and rapid cell-to-cell propagation of the virus, while others considered that discontinuing treatment could also worsen the general health status of patients and that maintaining treatment could have a favorable effect by acting as a vasodilator, antioxidant and anti-inflammatory through the action of Ang-(1-7) on MAS-receptors. Although we had not enough patients with HT medication to draw definitive conclusion, our preliminary observation suggests that being on HT medication could promote a faster return to normal ACE2 gene expression. It corroborates recent data indicating that maintaining therapy against HT improve the clinical outcome of COVID-19 patients (85–87).

The lower expression of ACE2 was consistent with the observed accumulation of Ang II in COVID-19 patients. Therefore, it may be surprising to find no major difference between the three groups studied with respect to the quantification of the Ang-(1-7) in plasma samples. In particular, we noted a higher Ang-(1-7) expression in three prolonged viral shedders, a 49-years-old woman with HT and the highest plasma concentrations of Ang II, a 81-years-old woman with HT and a high plasma concentration of sACE2, and a 52-years-old man with no known history of HT. A transcription of the ACE2 gene should result in a reduced capacity of this peptidase to cleave Ang II -. However, Ang-(1-7) production did not collapsed in COVID-19 patients and was slightly higher than in healthy volunteers. It cannot be excluded that ACE2 mRNA expression in PBMCs is valuable for this tissues only and that ACE2 may remain expressed on other cell types leading to cleavage of Ang II that is produced in excess in COVID-19 patients, allowing synthesis of Ang-(1-7) (88). However, it is much more likely that an alternative pathway independent of ACE2, contributes to the biosynthesis of Ang-(1-7). The comparison of intrarenal Ang I, Ang II and Ang-(1-7) in wild type mice and tisACE^-/-^ mice lacking the ACE, revealed that the Ang I and Ang II levels were decreased by 80% in tisACE^-/-^ mice, whereas the Ang-(1-7) levels were sustained (89). It was also reported that Ang-(1-7) can be formed directly from Ang I through peptidases including the endopeptidases neprilysin (NEP), a 95 kDa membrane-anchored metalloendopeptidase located on the vascular surface of blood vessels and the thimet oligopeptidase (TOP), a 80KDa soluble metalloendopeptidase, which both hydrolyzes Ang I to form Ang-(1-7) (90–92). This suggests that in COVID-19 patients the ACE2-independent pathway of peptide generation is efficient to trigger Ang-(1-7) biosynthesis from Ang I when the Ang-(1-7) production through Ang II cleavage by ACE2 is impaired. This should be further studied, as Ang-(1-7) generation was previously reported capable to achieve protection against Ang II-mediated lung injury and cardiovascular risks (84, 93, 94). However, the plasma concentration of Ang II in COVID-19 patients is probably too high so that its harmful effects are not offset by the protective action of Ang-(1-7). As previously reported (95), it is also likely that the high plasma concentration of Ang II triggers overproduction of aldosterone through AT1 receptor stimulation, which may be at least in part responsible of the hypokaliemia observed in some COVID-19 patients.

In conclusion, we report evidence that during the COVID-19, a decrease in the expression of ACE2 mRNA and cell-surface ACE2 is observed and that prolonged viral shedders forms of COVID-19 are associated with low plasma concentrations of sACE2. As a result, Ang II is no longer metabolized by ACE2 and its plasma concentrations increase. However, this has no direct impact on the plasma concentrations of Ang-(1-7) which remain stable in COVID-19 patients. This suggests that when the ACE2 pathway is less efficient or totally ineffective, Ang-(1-7) is produced by metabolism of Ang I likely by the neprilysin and/or thimet oligopeptidase. The high plasma concentration of Ang I in COVID-19 patients should favor this pathway. Although the plasma concentration of Ang-(1-7) remains stable in COVID-19 patients, it appears to be insufficient to prevent the harmful effects of Ang II.

## Data Availability Statement

The original contributions presented in the study are included in the article/**Supplementary Material**. Further inquiries can be directed to the corresponding author.

## Ethics Statement

The studies involving human participants were reviewed by French Participants Protection Committee (CPP East III; CPP president: Dr. P. Peton) prior to commencement of the study and received ethical approval CPP 20.04.09/N°:20.04.01.83219/N°7626. (Principal investigator, C. Devaux, Research Director at CNRS; Assay promotor: IHU Méditerranée Infection: Prof Didier Raoult, Director). The patients/participants provided their written informed consent to participate in this study.

## Author Contributions

IO, CM, DR, and CD contributed to the design of the study and conceived the manuscript. PB, LM, J-LM, and CD prepared the research protocol for ethical review by the French Participants Protection (CPP). IO performed the *in vitro* experiments. CM, PB, PP, AS, MM, and J-CL took care of the COVID-19 patients. CM prepared **Table 1**. IO prepared the figures. CD supervised the work and wrote the paper. DR obtained the funding for this study. All authors contributed to the article and approved the submitted version.

## Funding

This work was supported by the French Government under the « Investissements d’avenir » (Investments for the Future) program managed by the Agence Nationale de la Recherche (French ANR: National Agency for Research), (reference: Méditerranée Infection 10-IAHU-03), the Région Provence Alpes Côte d’Azur and European funding FEDER PRIMI.

## Conflict of Interest

CD declares owning Sanofi and Merck shares.

The remaining authors declare that the research was conducted in the absence of any commercial or financial relationships that could be construed as a potential conflict of interest.

## Supplementary Material

Supplementary Figure 1 Histogram summarizing the data of flow cytometry analysis of ACE2 expression at the surface of T-cells, B-cells and monocytes. Comparison of percent cells expressing ACE2 and ACE2 MFI on cell samples from prolonged viral shedders (n=12), patients short viral shedders (n=12) and healthy volunteers (n=12). The gating was performed using different cluster differentiation-specific mAb allowing to select subpopulation of lymphocytes CD3^+^ T-cells, CD20^+^ B-cells and HLA-DR^+^/CD16^+^ monocytic/dendritic cells. The left panels indicate the percent of cells expressing ACE2 with respect to the cells subpopulation analyzed while the right panels are the histograms of ACE2 cell surface expression (MFI).

Supplementary Figure 2 Correlation matrix (R studio used to generate the matrix) between sACE2 and angiotensin metabolites in different groups of COVID-19 patients and healthy volunteers. The upper panels show the correlation matrix of the different biomarkers of interest for the group of healthy volunteers (left) and prolonged viral shedders (right), while the correlation matrix for the short viral shedders is shown in the lower left panel. For the not normally distributed correlation (Healthy control and prolonged viral shedders) we used Spearman’s correlation and for the short viral shedders we used the Pearson’s correlation. The table at the lower right part of the figure summarizes the correlation analyses (Pearson test) between sACE2 and the different metabolites Ang I, Ang II, Ang-(1-7) in each group.

## References

[1] Zhou P Yang XL Wang XG Hu B Zhang L Zhang W. A Pneumonia Outbreak Associated With a New Coronavirus of Probable Bat Origin. Nature (2020) 579:270–3. 10.1038/s41586-020-2012-7 PMC7095418 32015507

[2] Afelt A Frutos R Devaux C. Bats, Coronaviruses, and Deforestation: Toward the Emergence of Novel Infectious Diseases? Front Microbiol (2018) 9:702. 10.3389/fmicb.2018.00702 29696007 PMC5904276

[3] Rota PA Oberste MS Monroe SS Nix WA Campagnoli R Icenogle JP. Characterization of a Novel Coronavirus Associated With Severe Acute Respiratory Syndrome. Science (2003) 300:1394–9. 10.1126/science.1085952 12730500

[4] Marra MA Jones SJ Astell CR Holt RA Brooks-Wilson A Butterfield YS. The Genome Sequence of the SARS-Associated Coronavirus. Science (2003) 300:1399–404. 10.1126/science.1085953 12730501

[5] Ge XY Li JL Yang XL Chmura AA Zhu G Epstein JH. Isolation and Characterization of a Bat SARS-Like Coronavirus That Uses the ACE2 Receptor. Nature (2013) 503:535–8. 10.1038/nature12711 PMC5389864 24172901

[6] Zhu N Zhang D Wang W Li X Yang B Song J. A Novel Coronavirus From Patients With Pneumonia in China, 2019. N. Engl J Med (2020) 382:727–33. 10.1056/NEJMoa2001017 PMC7092803 31978945

[7] Yang X Yu Y Xu J Shu H Xia JA Liu H. Clinical Course and Outcomes of Critically Ill Patients With SARS-Cov-2 Pneumonia in Wuhan, China: A Single-Centered, Retrospective, Observational Study. Lancet Resp Med (2020) 8(5):475–81. 10.1016/S2213-2600(20)30079-5 PMC7102538 32105632

[8] Frutos R Lopez Roig M Serra-Cobo J Devaux C. COVID-19: The Conjunction of Events Leading to the Pandemic and Lessons to Learn for Future Threats. Front Med (2020) 7:223. 10.3389/fmed.2020.00223 PMC7235412 32574324

[9] Huang C Wang Y Li X Ren L Zhao J Hu Y. Clinical Features of Patients Infected With 2019 Novel Coronavirus in Wuhan, China. Lancet (2020) 395(10223):497–506. 10.1016/S0140-6736(20)30183-5 31986264 PMC7159299

[10] Ksiazek TG Erdman D Goldsmith CS Zaki SR Peret T Emery S. A Novel Coronavirus Associated With Severe Acute Respiratory Syndrome. N Engl J Med (2003) 348:1953–66. 10.1056/NEJMoa030781 12690092

[11] Qin C Zhou L Hu Z Zhang S Yang S Tao Y. Dysregulation of Immune Response in Patients With COVID-19 in Wuhan, China. Clin Infect Dis (2020) 71(15):762–8. 10.1093/cid/ciaa248 PMC7108125 32161940

[12] Middeldorp S Coppens M van Haaps TF Foppen M Vlaar AP Müller MCA. Incidence of Venous Thromboembolism in Hospitalized Patients With COVID-19. J Thromb Haemost (2020) 18(8):1995–2002. 10.1111/jth.14888 32369666 PMC7497052

[13] Leonard-Lorant I Delabranche X Severac F Helms J Pauzet C Collange O. Acute Pulmonary Embolism in COVID-19 Patients on CT Angiography and Relationship to D-Dimer Levels. Radiology (2020) 296(3):E189–91. 10.1148/radiol.2020201561 PMC7233397 32324102

[14] Faggiano P Bonelli A Paris S Milesi G Bisegna S Bernardi N. Acute Pulmonary Embolism in COVID-19 Disease: Preliminary Report on Seven Patients. Int J Cardiol (2020) 313:129–31. 10.1016/j.ijcard.2020.04.028 PMC7250100 32471650

[15] Lippi G Favaloro EJ. D-Dimer is Associated With Severity of Coronavirus Disease 2019: A Pooled Analysis. Thromb Haemost (2020) 120(5):876–8. 10.1055/s-0040-1709650 PMC7295300 32246450

[16] Wang J Wang BJ Yang JC Wang MY Chen C Luo GX. Advances in the Research of Mechanism of Pulmonary Fibrosis Induced by Corona Virus Disease 2019 and the Corresponding Therapeutic Measures [Article in Chinese]. Zhonghua Shao Shang Za Zhi (2020) 36(8):691–7. 10.3760/cma.j.cn501120-20200307-00132 32174095

[17] Carsana L Sanzagni A Nasr A Rossi RS Pellegrinelli A Zerbi P. Pulmonary Post-Mortem Findings in a Series of COVID-19 Cases From Northern Italy: A Two-Centre Descriptive Study. Lancet Infect Dis (2020) 20(10):1135–40. 10.1016/S1473-3099(20)30434-5 PMC7279758 32526193

[18] AbdelMassih AF Kamel A Mishriky F Ismail HA El Qadi L Malak L. It is Infection or Rather Vascular Inflammation? Game-Changer Insights and Recommendations From Patterns of Multi-Organ Involvement and Affected Subgroups in COVID-19. Cardiovasc Endocrinol Metab (2020) 9(3):110–20. 10.1097/XCE.0000000000000211 PMC7410022 32803145

[19] Wang T Chen R Liu C Liang W Guan W Tang R. Attention Should be Paid to Venous Thromboembolism Prophylaxis in the Management of COVID-19. Lancet Haematol (2020) 7(5):e362–3. 10.1016/S2352-3026(20)30109-5 PMC7158946 32278361

[20] Tang N Bai H Chen X Gong J Li D Sun Z. Anticoagulant Treatment is Associated With Decreased Mortality in Severe Coronavirus Disease 2019 Patients With Coagulopathy. J Thromb Haemost (2020) 18(5):1094–9. 10.1111/jth.14817 PMC9906401 32220112

[21] Li W Zhang C Sui J Kuhn JH Moore MJ Luo S. Receptor and Viral Determinants of SARS-Coronavirus Adaptation to Human ACE2. EMBO J (2005) 24:1634–43. 10.1038/sj.emboj.7600640 PMC1142572 15791205

[22] Yan R Zhang Y Li Y Xia L Guo Y Zhou Q. Structural Basis for the Recognition of the SARS-Cov-2 by Full-Length Human ACE2. Science (2020) 367(6485):1444–8. 10.1126/science.abb2762 PMC7164635 32132184

[23] Qiu Y Zhao YB Wang Q Li JY Zhou ZJ Liao CH. Predicting the Angiotensin Converting Enzyme 2 (ACE2) Utilizing Capability as the Receptor of SARS-Cov-2. Microbes Infect (2020) 22(4-5):221–5. 10.1016/j.micinf.2020.03.003 PMC7156207 32199943

[24] Hamming I Timens W Bulthuis M Lely T Navis G van Goor H. Tissue Distribution of ACE2 Protein, the Functional Receptor for SARS Coronavirus. J Pathol (2004) 203(2):631–7. 10.1002/path.1570 PMC7167720 15141377

[25] Zhao Y Zhao Z Wang Y Zhou Y Ma Y Zuo W. Single-Cell RNA Expression Profiling of ACE2, the Receptor of SARS-Cov-2. Am J Resp Crit Care Med (2020) 202(5):756–9. 10.1101/2020.01.26.919985 PMC7462411 32663409

[26] Lamers MM Beumer J van der Vaart J Knoops K Puschhof J Breugem TI. SARS-Cov-2 Productively Infects Human Gut Enterocytes. Science (2020) 369(6499):50–4. 10.1126/science.abc1669 PMC7199907 32358202

[27] Devaux CA Lagier J-C and Raoult D. New Insights Into the Physiopathology of COVID-19: SARS-Cov-2-Associated Gastrointestinal Illness. Front Med (2021) 8:640073. 10.3389/fmed.2021.640073 PMC7930624 33681266

[28] Ferrario CM Chappell MC Tallant EA Brosnihan KB Diz DI. Counterregulatory Actions of Angiotensin-(1–7). Hypertension (1997) 30:535–41. 10.1161/01.HYP.30.3.535 9322978

[29] Tipnis SR Hooper NM Hyde R Karran E Christie G Turner AJ. A Human Homolog of Angiotensin-Converting Enzyme. Cloning and Functional Expression as a Captopril-Insensitive Carboxypeptidase. J Biol Chem (2000) 275(43):33238–43. 10.1074/jbc.m002615200 10924499

[30] Turner AJ. Exploring the Structure and Function of Zinc Metallopeptidases: Old Enzymes and New Discoveries. Biochem Soc Trans (2003) 31(Pt3):723–7. 10.1042/bst0310723 12773192

[31] Oudit GY Crackower MA Backx PH Penninger JM. The Role of ACE2 in Cardiovascular Physiology. Trends Cardiovasc Med (2003) 13(3):93–101. 10.1016/s1050-1738(02)00233-5 12691672

[32] Tallant EA Clark MA. Molecular Mechanisms of Inhibition of Vascular Growth by Angiotensin-(1-7). Hypertension (2003) 42(4):574–9. 10.1161/01.HYP.0000090322.55782.30 12953014

[33] Donoghue M Hsieh F Baronas E Godbout K Gosselin M Stagliano N. A Novel Angiotensin-Converting Enzyme-Related Carboxypeptidase. (ACE2) Converts Angiotensin I to Angiotensin 1–9. Circ Res (2000) 87(5):E1–9. 10.1161/01.res.87.5.e1 10969042

[34] Pena Silva RA Chu Y Miller JD Mitchell IJ Penninger JM Faraci FM. Impact of ACE2 Deficiency and Oxidative Stress on Cerebrovascular Function With Aging. Stroke (2012) 43(12):3358–63. 10.1161/STRKEAHA.112.667063 PMC3529166 23160880

[35] Lovren F Pan Y Quan A Teoh H Wang G Shukla PC. Angiotensin Converting Enzyme-2 Confers Endothelial Protection and Attenuates Atherosclerosis. Am J Physiol Heart Circ Physiol (2008) 295(4):H1377–84. 10.1152/&jpheart.00331.2008 18660448

[36] Haschke M Schuster M Poglitsch M Loiner H Salzberg M Bruggisser M. Pharmacokinetics and Pharmacodynamics of Recombinant Human Angiotensin-Converting Enzyme 2 in Healthy Human Subjects. Clin Pharmacokinet (2013) 52:783–92. 10.1007/s40262-013-0072-7 23681967

[37] Zoufaly A Poglitsch M Aberle JH Hoepler W Seitz T Traugott M. Human Recombinant Soluble ACE2 in Severe COVID-19. Lancet Resp Med (2020) 8:115–58. 10.1016/S2213-2600(20)30418-5 PMC7515587 33131609

[38] Glowacka I Bertram S Herzog P Pfefferle S Steffen I Muench MO. Differential Downregulation of ACE2 by the Spike Proteins of Severe Acute Respiratory Syndrome Coronavirus and Human Coronavirus NL63. J Virol (2010) 84(2):1198–205. 10.1128/JVI.01248-09 PMC2798380 19864379

[39] Kuba K Imai Y Rao S Gao H Guo F Guan B. A Crucial Role of Angiotensin Converting Enzyme 2 (ACE2) in SARS Coronavirus–Induced Lung Injury. Nat Med (2005) 11:875–9. 10.1038/nm1267 PMC7095783 16007097

[40] Zhuang M-W Cheng Y Zhang J Jiang X-M Wang L Deng J. Increasing Host Cellular Receptor—Angiotensin Converting Enzyme 2 (ACE2) Expression by Coronavirus may Facilitate 2019-Ncov (or SARS-Cov-2) Infection. J Med Virol (2020) 92(11):2693–701. 10.1002/jmv.26139 PMC7300907 32497323

[41] Devaux CA Rolain JM Raoult D. ACE2 Receptor Polymorphism: Susceptibility to SARS-Cov-2, Hypertension, Multi-Organ Failure, and COVID-19 Disease Outcome. J Microbiol Immunol Infect (2020) 53(3):425–35. 10.1016/j.jmii.2020.04.015 PMC7201239 32414646

[42] La Scola B Le Bideau M Andreani J Hoang VT Grimaldier C Colson P. Viral RNA Load as Determined by Cell Culture as a Management Tool for Discharge of SARS-Cov-2 Patients From Infectious Disease Wards. Eur J Clin Microbiol Infect Dis (2020) 39(6):1059–61. 10.1007/s10096-020-03913-9 PMC7185831 32342252

[43] WHO. Coronavirus Disease 2019, Case Definitions. Available at: https://www.who.int/docs/default-source/coronaviruse/situation-reports/20200321-sitrep-61-covid-19.pdf.

[44] Gautret P Lagier JC Parola P Hoang VT Meddeb L Mailhe M. Hydroxychloroquine and Azithromycin as a Treatment of COVID-19: Results of an Open-Label non-Randomized Clinical Trial. Int J Antimicrob Agents (2020) 56(1):105949. 10.1016/j.ijantimicag.2020.105949 32205204 PMC7102549

[45] Gautret P Million M Jarrot PA Camoin-Jau L Colson P Fenollar F. Natural History of COVID-19 and Therapeutic Options. Expert Rev Clin Immunol (2020) 16(12):1159–84. 10.1080/1744666X.2021.1847640 33356661

[46] Chappell MC Pirro NT South AM Gwathmey TYM. Concerns on the Specificity of Commercial Elisas for the Measurement of Angiotensin (1-7) and Angiotensin II in Human Plasma. Hypertension (2021) 77:e29–31. 10.1161/HYPERTENSIONAHA.120.16724 PMC7878344 33399002

[47] Sparks MA South AM Badley AD Baker-Smith CM Battle D Bozkurt B. Severe Acute Respiratory Syndrome Coronavirus 2, COVID-19, and the Renin-Angiotensin System Pressing Needs and Best Research Practices. Hypertension (2020) 76(5):1350–67. 10.1161/HYPERTENSIONAHA.120.15948 PMC7685174 32981369

[48] Yan R Zhang Y Li Y Xia L Guo Y Zhou Q. Structural Basis for the Recognition of the SARS-Cov-2 by Full-Length Human ACE2. Science (2020) 367(6485):1444–8. 10.1126/science.abb2762 PMC7164635 32132184

[49] Wrapp D Wang N Corbett KS Goldsmith JA Hsieh CL Abiona O. Cryo-EM Structure of the 2019-Ncov Spike in the Prefusion Conformation. Science (2020) 367(6483):1260–3. 10.1126/science.abb2507 PMC7164637 32075877

[50] Devaux C Pinault L Omar Osman I Raoult D. Can ACE2 Receptor Polymorphism Predict Species Susceptibility to SARS-Cov2? Front Public Health (2021) 8:608765. 10.3389/fpubh.2020.608765 33643982 PMC7902720

[51] AbdelMassih AF Kamel A Mishriky F Ismail HA Qadi El Malak L. et al. Is it Infection or Rather Vascular Inflammation? Game-Changer Insights and Recommendations From Patterns of Multi-Organ Involvement and Affected Subgroups in COVID-19. Cardiovasc Endocrinol Metabol (2020) 9(3):110–20. 10.1097/XCE.0000000000000211 PMC7410022 32803145

[52] Guy JL Jackson RM Jensen HA Hooper NM Turner AJ. Identification of Critical Active-Site Residues in Angiotensin-Converting Enzyme-2 (ACE2) by Site-Directed Mutagenesis. FEBS J (2005) 272(14):3512–20. 10.1111/j.1742-4658.2005.04756.x PMC7164114 16008552

[53] Rutkowska-Zapala M Suski M Szatanek R Lenart M Weglarczyk K Olszanecki R. Human Monocyte Subsets Exhibit Divergent Angiotensin I-Converting Activity. Clin Exp Immunol (2015) 181(1):126–32. 10.1111/cei.12612 PMC4469162 25707554

[54] Sun P Lu X Xu C Sun W Pan B. Understanding of COVID-19 Based on Current Evidence. J Med Virol (2020) 92(6):548–51. 10.1002/jmv.25722 PMC7228250 32096567

[55] Xu H Zhong L Deng J Peng J Dan H Zeng X. High Expression of ACE2 Receptor of 2019-Ncov on the Epithelial Cells of Orla Mucosa. Int J Oral Sci (2020) 12:8. 10.1038/s41368-020-0074-x 32094336 PMC7039956

[56] Hashimoto T Perlot T Rehman A Trichereau J Ishiguro H Paolino M. ACE2 Links Amino Acid Malnutrition to Microbial Ecology and Intestinal Inflammation. Nature (2012) 487:477–81. 10.1038/nature11228 PMC7095315 22837003

[57] Dai S Ding M Liang N Zhuo Li Li D Guan L. Associations of ACE I/D Polymorphism With the Levels of ACE, Kallikrein, Angiotensin II and Interleukin-6 in STEMI Patients. Sci Rep (2019) 9:19719. 10.1038/s41598-019-56263-8 31873176 PMC6927979

[58] Delanghe JR Speeckaert MM De Buyze ML. COVID-19 Infections are Also Affected by Human ACE1 D/I Polymorphism. Clin Chem Lab Med (2020) 58(7):1125–6. 10.1515/cclm-2020-0425 32286246

[59] Hatami N Ahi S Sadeghinikoo A Foroughian M Javdani F Kalani N. Worldwide ACE(I/D) Polymorphism may Affect COVID-19 Recovery Rate: An Ecological Meta-Regression. Endocrine (2020) 68:479–84. 10.1007/s12020-020-02381-7 PMC7294766 32542429

[60] Chen Q Tang X Yu CQ Chen DA Tian J Cao Y. Correlation of Angiotensin-Converting Enzyme 2 Gene Polymorphism With Antihypertensive Effects of Benazepril. Beijing Da Xue Xue Bao (2010) 42(3):293–8. 20559404

[61] Luo Y Liu C Guan T Li Y Lai Y Li F. Association of ACE2 Genetic Polymorphisms With Hypertension-Related Target Organ Damages in South Xinjiang. Hypertens Res (2019) 42(5):681–9. 10.1038/s41440-018-0166-6 PMC6477792 30542083

[62] Cao Y Li L Feng Z Wan S Huang P Sun X. Comparative Genetic Analysis of the Novel Coronavirus (2019-Ncov/SARS-Cov-2) Receptor ACE2 in Different Populations. Cell Discovery (2020) 6:11. 10.1038/s41421-020-0147-1 32133153 PMC7040011

[63] Dai YJ Hu F Li H Huang HY Wang DW Liang Y. A Profiling Analysis on the Receptor ACE2 Expression Reveals the Potential Risk of Different Type of Cancers Vulnerable to SARS-Cov-2 Infection. Ann Transl Med (2020) 8(7):481. 10.21037/atm.2020.03.61 32395525 PMC7210193

[64] Gwathmey TM Shaltout HA Nixon PA O’Shea TM Rose JC Washburn LK. Gender Differences in Urinary ACE and ACE2 Activities in Adolescents. FASEB J (2008) 22(S1):940. 10.1096/fasebj.22.1_supplement.940.6

[65] Devaux CA Rolain JM Colson P Raoult D. New Insights on the Antiviral Effects of Chloroquine Against Coronavirus: What to Expect for COVID-19? Int J Antimicrob Agents (2020) 55(5):105938. 10.1016/j.ijantimicag.2020.105938 32171740 PMC7118659

[66] Fantini J Di Scala C Chahinian H Yahi N. Structural and Molecular Modelling Studies Reveal a New Mechanism of Action of Chloroquine and Hydroxychloroquine Against SARS-Cov-2 Infection. Int J Antimicrob Agents (2020) 55(5):105960. 10.1016/j.ijantimicag.2020.105960 32251731 PMC7128678

[67] Tikellis C Pickering R Tsorotes D Du XJ Kiriazis H Nguyen-Huu T-P. Interaction of Diabetes and ACE2 in the Pathogenesis of Cardiovascular Disease in Experimental Diabetes. Clin Sci (Lond) (2012) 123(8):519–29. 10.1042/CS20110668 22616805

[68] Velkoska E Patel SK Burrell LM. Angiotensin Converting Enzyme 2 Anddiminazene: Role in Cardiovascular and Blood Pressure Regulation. Curr Opin Nephrol Hypertens (2016) 25(5):384–95. 10.1097/MNH.0000000000000254 27367913

[69] Gallagher PE Ferrario CM Tallant EA. Regulation of ACE2 in Cardiac Myocytes and Fibroblasts. Am J Physiol Heart Circ Physiol (2009) 295(6):H2373–9. 10.1152/ajpheart.00426.2008 PMC2614534 18849338

[70] Carvelli J Demaria O Vély F Batista L Chouaki Benmansour N Fares J. Association of COVID-19 Inflammation With Activation of the C5a-C5ar1axis. Nature (2020) 588:146–50. 10.1038/s41586-020-2600-6 PMC7116884 32726800

[71] Lambert D Yarski M Warner FJ Thornhill P Parkin ET Smith AI. Tumor Necrosis Factor- Convertase (ADAM17) Mediates Regulated Ectodomain Shedding of the Severe-Acute Respiratory Syndrome-Coronavirus (SARS-Cov) Receptor, Angiotensin-Converting Enzyme-2 (ACE2). J Biol Chem (2005) 280(34):30113–9. 10.1074/jbc.M505111200 PMC8062222 15983030

[72] Glowacka I Bertram S Herzog P Pfefferle S Steffen I Muench MO. Differential Downregulation of ACE2 by the Spike Proteins of Severe Acute Respiratory Syndrome Coronavirus and Human Coronavirus NL63. J Virol (2010) 84(2):1198–205. 10.1128/JVI.01248-09 PMC2798380 19864379

[73] Patel VB Clarke N Wang Z Fan D Parajuli N Basu R. Angiotensinii Induced Proteolytic Cleavage of Myocardial ACE2 is Mediated by TACE/ADAM-17: A Positive Feedback Mechanism in the RAS. J Mol Cell Cardiol (2014) 66:167–76. 10.1016/j.yjmcc.2013.11.017 24332999

[74] Brojakowska A Narula J Shimony R Bander J. Clinical Implications of SARS-Cov-2 Interaction With Renin Angiotensin System. J Am Col Cardiol (2020) 75(24):3085–95. 10.1016/j.jacc.2020.04.028 PMC7161517 32305401

[75] Burrell LM Harrap SB Velkoska E Patel SK. The ACE2 Gene: Its Potential as a Functional Candidate for Cardiovascular Disease. Clin Sci (Lond) (2013) 124(2):65–76. 10.1042/CS20120269 23013041

[76] Devaux CA. Are ACE Inhibitors and Arbs More Beneficial Than Harmful in the Treatment of Severe COVID-19 Disease? J Cardiovasc Med Cardio (2020) 7(2):101–3. 10.17352/2455-2976.000122

[77] Bavishi C Maddox TM Messerli FH. Coronavirus Disease 2019 (COVID-19) Infection and Renin Angiotensin System Blockers. JAMA Cardiol (2020) 5(7):745–7. 10.1001/jamacardio.2020.1282 32242890

[78] Pires de Souza GA Omar Osman I Le Bideau M Baudoin JP Jaafar R Devaux CA. Angiotensin II Receptor Blockers (Arbs Antihypertensive Agents) Increase Replication of SARS-Cov-2 in Vero E6 Cells. Front Cell Infect Microbiol (2021). 10.3389/fcimb.2021.639177 PMC8231006 34178717

[79] Wysocki J Lores E Minghao Ye MJ S Batlle D. Kidney and Lung ACE2 Expression After an ACE Inhibitor or an Ang II Receptor Blocker: Implications for COVID-19. JASN (2020) 31:1941–3. 10.1681/ASN.2020050667 PMC7461678 32669323

[80] Milne S Yang CX Timens W Bossé Y Sin DD. SARS-Cov-2 Receptor ACE2 Gene Expression and RAAS Inhibitors. Lancet Resp Med (2020) 8(6):E50–1. 10.1016/S2213-2600(20)30224-1 PMC7220165 32411576

[81] Fang L Karakiulakis G Roth M. Antihypertensive Drugs and Risk of COVID-19? Lancet Resp Med (2020) 8(5):E32–3. 10.1016/S2213-2600(20)30159-4 PMC7194912 32222169

[82] Patel AB. And Verma a. COVID-19 and Angiotensin-Converting Enzyme Inhibitors and Angiotensin Receptor Blockers: What is the Evidence? JAMA (2020) 323(18):1769–70. 10.1001/jama.2020.4812 32208485

[83] Vaduganathan M Vardeny O Michel T McMurray JJV Pfeffer MA Soloman SD. Renin-Angiotensin-Aldosterone System Inhibitors in Patients With Covid-19. New Engl J Med (2020) 382:1653–9. 10.1056/NEJMsr2005760 PMC7121452 32227760

[84] Fang L Karakiulakis G Roth M. Are Patients With Hypertension and Diabetes Mellitus at Increased Risk for COVID-19 Infection? Lancet (2020) 8(4):E21. 10.1016/S2213-2600(20)30116-8 PMC7118626 32171062

[85] Meng J Xiao G Zhang J He X Ou M Bi J. Renin-Angiotensin System Inhibitors Improve the Clinical Outcomes of COVID-19 Patients With Hypertension. Emerg Microb Infect (2020) 9(1):757–60. 10.1080/22221751.2020.1746200 PMC7170368 32228222

[86] Bean DM Kraljevic Z Searle T Bendayan R O’Gallagher K Pickles A. Angiotensin-Converting Enzyme Inhibitors and Angiotensin II Receptor Blockers are Not Associated With Severe COVID-19 Infection in a Multi-Site UK Acute Hospital Trust. Eur J Heart Fail (2020) 22(6):967–74. 10.1002/ejhf.1924 PMC7301045 32485082

[87] Yang G Tan Z Zhou L Yang M Peng L Liu J. Effects of Angiotensin II Receptor Blockers and ACE (Angiotensin-Converting Enzyme) Inhibitors on Virus Infection, Inflammatory Status, and Clinical Outcomes in Patients With COVID-19 and Hypertension: A Single-Center Retrospective Study. Hypertension (2020) 76(1):51–8. 10.1161/HYPERTENSIONAHA.120.15143 32348166

[88] Li MY Li L Zhang Y Wang XS. Expression of the SARS-Cov2 Cell Receptor Gene *ACE2* in a Wide Variety of Human Tissues. Infect Dis Poverty (2020) 9:45. 10.1186/s40249-020-00662-x 32345362 PMC7186534

[89] Modrall JG Sadjadi J Brosnihan KB Gallagher PE Yu CH Kramer GL. Depletion of Tissue Angiotensin-Converting Enzyme Differentially Influences the Intrarenal and Urinary. Hypertension (2004) 43(4):849–53. 10.1161/01.HYP.0000121462.27393.f6 14981053

[90] Rice GI Thomas DA Grant PJ Turner AJ Hooper NM. Evaluation of Angiotensin-Converting Enzyme (ACE), its Homologue ACE2 and Neprilysin in Angiotensin Peptide Metabolism. Biochem J (2004) 383(Pt 1):45–51. 10.1042/BJ20040634 15283675 PMC1134042

[91] Pereira MG Souza LL Becari C Duarte DA Camacho FR Oliveira JA. Angiotensin II-Independent Angiotensin-(1-7) Formation in Rat Hippocampus: Involvement of Thimet Oligopeptidase. Hypertension (Dallas TX: 1979) (2013) 62(5):879–85. 10.1161/HYPERTENSIONAHA.113.01613 24041943

[92] Chappell MC. The Angiotensin-(1-7) Axis: Formation and Metabolism Pathways. In: Santos R, editor. Angiotensin-(1-7). Switzerland: Springer Nature (2019). p. 1–26. 10.1007/978-3-030-22696-1_1

[93] Forrester SJ Booz GW Sigmund CD Coffman TM Kawai T Rizzo V. Angiotensin II Signal Transduction: An Update on Mechanisms of Physiology and Pathophysiology. Physiol Rev (2018) 98(3):1627–738. 10.1152/physrev.00038.2017 PMC6335102 29873596

[94] Brasier AR Recinos A III Eledrisi MS. Vascular Inflammation and the Renin-Angiotensin System. Arterioscler Thromb Vasc Biol (2002) 22:1257–66. 10.1161/01.ATV.0000021412.56621.A2 12171785

[95] Alfano G Ferrari A Fontana F Perrone R Mori G Ascione E. Hypokalemia in Patients With COVID-19. Clin Exp Nephrol (2021) 4:1–9. 10.1007/s10157-020-01996-4 PMC7781399 33398605

