# Supplementary figures and images for: Expression of ACE2, Soluble ACE2, Angiotensin I, Angiotensin II and Angiotensin-(1-7) Is Modulated in COVID-19 Patients
Source: Front Immunol. 2021 Jun 14;12:625732. doi: 10.3389/fimmu.2021.625732 (PMC8236950; doi:10.3389/fimmu.2021.625732)

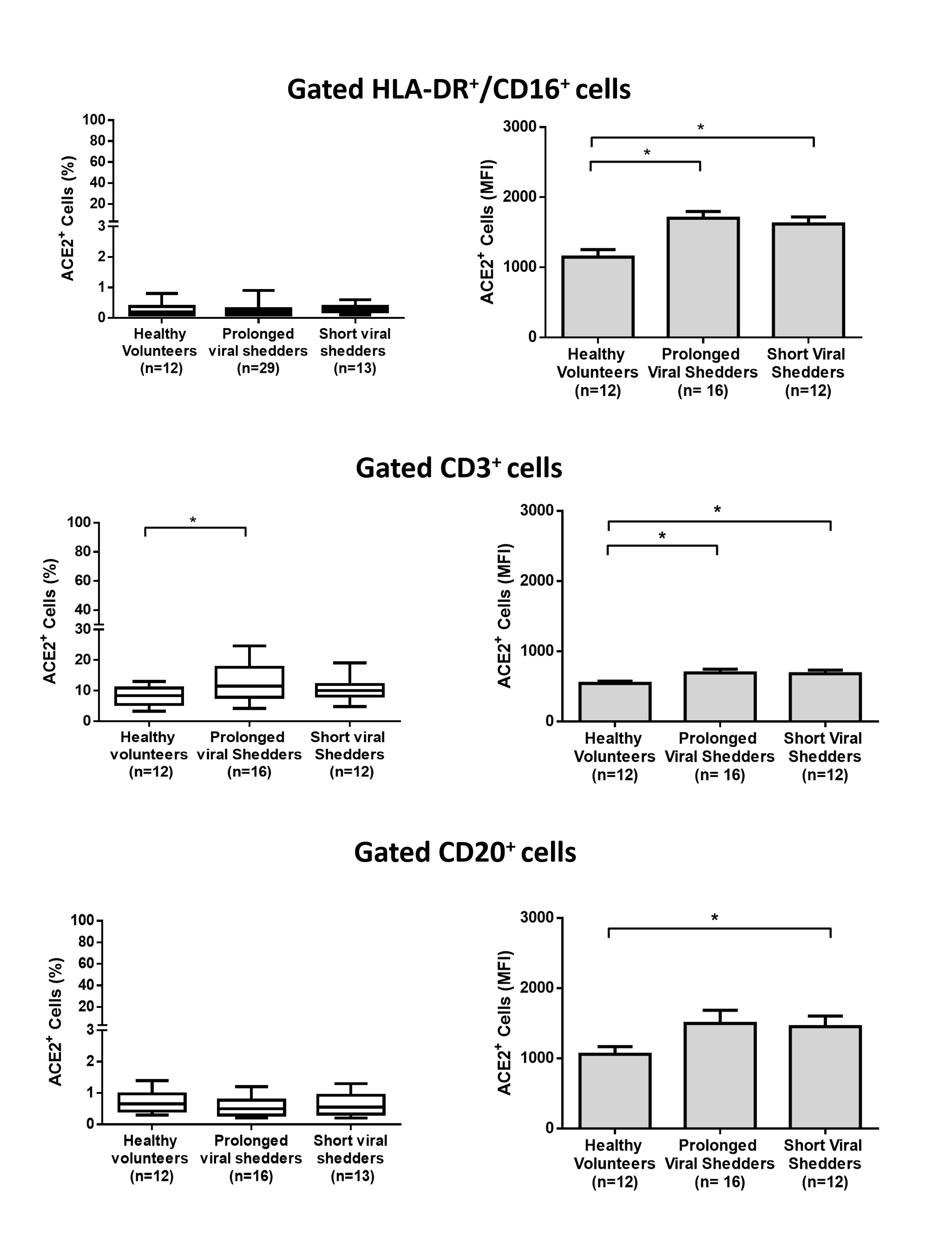

Supplement: Supplementary Figure 1 — Histogram summarizing the data of flow cytometry analysis of ACE2 expression at the surface of T-cells, B-cells and monocytes. Comparison of percent cells expressing ACE2 and ACE2 MFI on cell samples from prolonged viral shedders (n=12), patients short viral shedders (n=12) and healthy volunteers (n=12). The gating was performed using different cluster differentiation-specific mAb allowing to select subpopulation of lymphocytes CD3+ T-cells, CD20+ B-cells and HLA-DR+/CD16+ monocytic/dendritic cells. The left panels indicate the percent of cells expressing ACE2 with respect to the cells subpopulation analyzed while the right panels are the histograms of ACE2 cell surface expression (MFI). [file Image_1.tif]

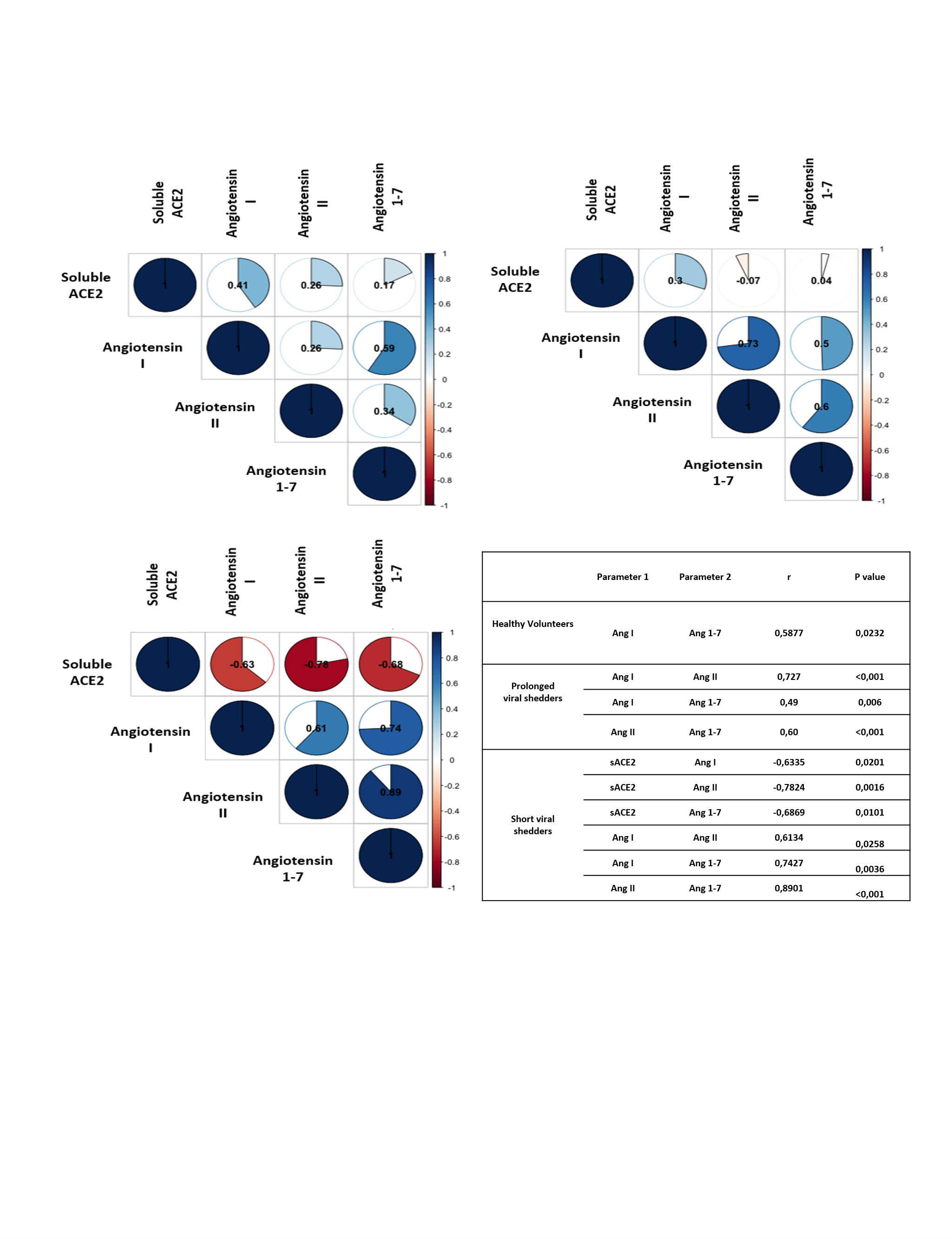

Supplement: Supplementary Figure 2 — Correlation matrix (R studio used to generate the matrix) between sACE2 and angiotensin metabolites in different groups of COVID-19 patients and healthy volunteers. The upper panels show the correlation matrix of the different biomarkers of interest for the group of healthy volunteers (left) and prolonged viral shedders (right), while the correlation matrix for the short viral shedders is shown in the lower left panel. For the not normally distributed correlation (Healthy control and prolonged viral shedders) we used Spearman’s correlation and for the short viral shedders we used the Pearson’s correlation. The table at the lower right part of the figure summarizes the correlation analyses (Pearson test) between sACE2 and the different metabolites Ang I, Ang II, Ang-(1-7) in each group. [file Image_2.tif]
